# Identification of potential neuromotor mechanisms of manual therapy in patients with musculoskeletal disablement: rationale and description of a clinical trial

**DOI:** 10.1186/1471-2377-9-20

**Published:** 2009-05-21

**Authors:** Beth E Fisher, Todd E Davenport, Kornelia Kulig, Allan D Wu

**Affiliations:** 1 Neuroplasticity and Imaging Laboratory, Division of Biokinesiology and Physical Therapy at the School of Dentistry, University of Southern California, Los Angeles, California, USA; 2 Department of Physical Therapy, Thomas J. Long School of Pharmacy and Health Sciences, University of the Pacific, Stockton, California, USA; 3 Musculoskeletal Biomechanics Research Laboratory, Division of Biokinesiology and Physical Therapy at the School of Dentistry, University of Southern California, Los Angeles, California, USA; 4 Department of Neurology, David Geffen School of Medicine, University of California Los Angeles, Los Angeles, California, USA

## Abstract

**Background:**

Many health care practitioners use a variety of hands-on treatments to improve symptoms and disablement in patients with musculoskeletal pathology.

Research to date indirectly suggests a potentially broad effect of manual therapy on the neuromotor processing of functional behavior within the supraspinal central nervous system (CNS) in a manner that may be independent of modification at the level of local spinal circuits. However, the effect of treatment speed, as well as the specific mechanism and locus of CNS changes, remain unclear.

**Methods/Design:**

We developed a placebo-controlled, randomized study to test the hypothesis that manual therapy procedures directed to the talocrural joint in individuals with post-acute ankle sprain induce a change in corticospinal excitability that is relevant to improve the performance of lower extremity functional behavior.

**Discussion:**

This study is designed to identify potential neuromotor changes associated with manual therapy procedures directed to the appendicular skeleton, compare the relative effect of treatment speed on potential neuromotor effects of manual therapy procedures, and determine the behavioral relevance of potential neuromotor effects of manual therapy procedures.

**Trial Registration:**

identifier NCT00847769.

## Background

Many health care practitioners use a variety of hands-on treatments to improve symptoms and disablement in patients with musculoskeletal pathology. Often, these manual procedures purport to target specific musculoskeletal structures, such as joints, muscles, or fascia. Procedures that historically have been relevant to joint structures may be placed on a continuum of speed and amplitude. Mounting scientific evidence supports statistically significant and clinically important benefits of manual therapy in subgroups of patients with various forms of musculoskeletal disablement affecting the axial region [1-3] and appendicular region. [4-9] However, studies related to the mechanisms of manual therapy interventions have lagged behind the literature documenting their clinical effects. An improved understanding of the mechanism of clinical improvement with various speeds and amplitudes of manual therapy will lead to the optimization of patient selection for manual therapy procedures. In turn, improved patient selection will optimize efficiency, quality, and cost of health care for patients with musculoskeletal disablement.

Various central and spinal sensorimotor mechanisms of manual therapy procedures recently have been investigated. Inhibition of the Hoffman reflex following spinal manipulation and increased lower extremity muscle strength have been observed following manual therapy directed to the lumbopelvic region in several studies. [10-14] Manual therapy procedures may facilitate descending GABAergic inputs to local spinal circuits that cause the observed H-reflex depression, suggesting a broader effect on the central nervous system (CNS).[15] However, relatively few studies to date have looked into short-term neuroplastic changes in CNS neuromotor processing to manual therapy procedures. To that extent, Dishman and colleagues[16] identified a short-term increase in motor evoked potential amplitude for the lumbar paraspinals in healthy volunteers using single-pulse transcranial magnetic stimulation (TMS) directed to contralateral motor cortex. Haavik-Taylor and Murphy[17] also documented a significant muscle-specific pattern of effects of cervical spine manipulation on short interval intracortical facilitation, short interval intracortical inhibition, and cortical silent period of abductor pollicis brevis and extensor indicis without significant change in F wave in asymptomatic individuals with a history of recurrent neck pain.

Taken together, these results suggest a potentially broad effect of manual therapy on the neuromotor processing of functional behavior within the supraspinal CNS in a manner that may be independent of modification at the level of local spinal circuits. However, several important limitations of existing studies continue to constrain our collective understanding of the CNS changes associated with the clinical effects of manual therapy in patients with musculoskeletal disablement. The use of non-disabled volunteers in the majority of existing research to date may provide limited information regarding the specific effects of mobilization and manipulation in patients with disablement due to pain and weakness. The use of spinal manual therapy as a subject of study potentially jeopardizes the specificity of conclusions that can be drawn, since spinal manipulation is poorly localized even in skilled and experienced practitioners.[18] The absence in the literature to date of behavioral measures to document potential changes in physical performance as a result of manual therapy procedures means the functional relevance of observed CNS neuroplasticity associated with cervical and lumbar manipulation also remains unclear. Also, the effect of procedure speed on CNS neuroplasticity has yet to be examined. A comparison of procedures characterized by a high velocity-low amplitude (HVLA) application of iatrogenic force with procedures that involve a slower, variable-amplitude application of iatrogenic force would be relevant, because they are among the most common clinical procedures in manual therapy.

One promising research design for determining the underlying neural mechanism associated with manual therapy involves the talocrural joint. There are a number of advantages for using the talocrural joint as an experimental preparation for studying the neurophysiological effects of manual therapy procedures. These include (i) the relatively large size of the talocrural joint, (ii) the relatively large size of the muscle groups crossing this joint, and (iii) the talocrural joint is subject to a prevalent injury that may be identified on the basis of a simple history and clinical examination. The large size of the talocrural joint suggests manual therapy procedures may be more specifically directed to this region than a smaller joint of the spine. The relatively large size of the muscles crossing the talocrural joint provide for easily reproducible recording sites for transcranial magnetic stimulation with electromyographic recording (TMS/EMG). Valid and reliable behavioral measurements for talocrural joint range of motion and lower extremity functional behavior exist. These measurements will allow for empirical examination of the relationship between short-term CNS neuroplasticity and the changes in functional behavior that have been elucidated by clinical studies.

The purpose of this paper is to describe methodology for using the talocrural joint as an experimental preparation to study the neuroplastic CNS changes associated with mobilization and manipulation. The specific aims of this study are to (1) determine the same-day test-retest reliability of TMS/EMG in individuals with post-acute ankle sprains, (2) quantify corticospinal excitability in individuals with post-acute ankle sprains receiving HVLA ankle manual therapy, slow ankle manual therapy, and control intervention; and (3) relate changes in corticospinal excitability in individuals with post-acute ankle sprains receiving HVLA ankle manual therapy, slow ankle manual therapy, and control intervention to short-term changes in lower extremity function.

## Methods/Design

### Subjects

Subjects with post-acute ankle sprains (n = 27) will be enrolled into the study. Inclusion criteria include (i) subject age between 18–60 years, (ii) onset of ankle sprain at least 2 weeks prior to enrollment, (iii) Foot and Ankle Ability Measure Activity of Daily Living subscale score >20%, and (iv) ankle dorsiflexion range of motion limitation greater than 2 standard deviations from published values.[19] Exclusion criteria include (i) assisted ambulation (eg, cane or crutches); (ii) inability to bear weight through the affected extremity immediately after injury; (iii) tenderness to palpation of the medial and lateral malleolar zones, styloid process of the 5th metatarsal, and navicular[20]; (iv) positive anterior drawer or talar tilt dimple test suggesting ligamentous laxity [21-23]; (v) volume of the affected limb greater than 10% of the unaffected limb per water displacement volumetry[24]; (vi) previous history of ligament or bony reconstructive surgery to the ankle and foot; (vii) concomitant injury to other lower extremity joints; and (viii) medical conditions that serve as contraindications to mobilization/manipulation and transcranial magnetic stimulation, such as presence of pacemaker, metal in head, pregnancy, neurological disorders, recent use of stimulants or medications known to lower seizure threshold, and personal or family history of seizures.[25,26]

A total of 27 subjects will be recruited for this study (n = 9 per group). This sample size will provide >80% statistical power to detect differences in lateral star balance excursion performance based on an effect size d = 1.47 from Hale and colleagues[27] involving change in SBET score due to an exercise-based rehabilitation program in individuals with ankle sprains. Therefore, this study was powered to detect between-group differences in the selected lower extremity functional behavior following talocrural manipulation, which is thought to be the dependent variable in this study that will demonstrate the greatest intersubject variability.

### Research Design

Subjects each begin with pre-intervention TMS measures (motor thresholds and input-output curves), ankle range of motion (ROM) tests, and lower extremity functional behavior tests. Each pre-intervention measurement will be taken a total of 2 times with a 30 minute rest break between measurements. Subjects will then be randomized into 1 of 3 comparison groups, each to receive a different manual therapy intervention. Immediately following the intervention, subjects will receive post-intervention TMS measures, ankle ROM tests, and lower extremity functional behavior tests, identical to the pre-intervention assessments.

### Procedure

**Figure 1:**
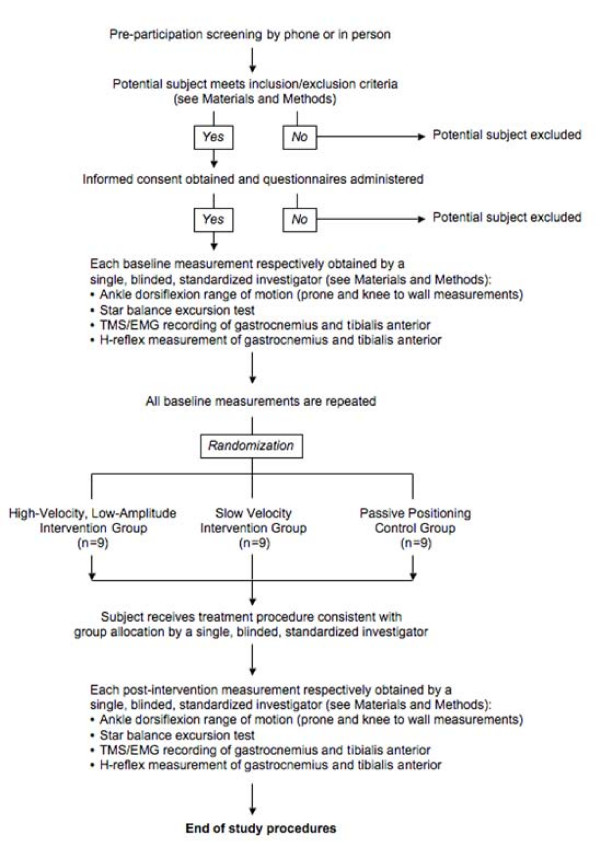
**Flow diagram for subjects' screening, testing, and intervention**.

All subjects who meet the inclusion and exclusion criteria will be eligible for participation (Figure 1). Potential subjects also will be screened prior to enrollment into this study to determine presence of decreased ankle dorsiflexion range of motion (ROM) greater than 2 standard deviations from published norms for standard procedures for range of motion.[19] Upon arrival on the day of testing, participants will complete a 15-minute intake interview that includes questions about their medical and psychiatric history, and their current use of alcohol, nicotine and prescription or non-prescription drugs. The purpose of the interview is to obtain more detailed information about any factors that may influence either their eligibility to participate in the TMS/EMG procedures or the results of the TMS/EMG measurements themselves. Patients will then receive pre-intervention testing, randomization into intervention groups and study-related intervention, and post-intervention testing. Study procedures will last up to 3 hours. The Institutional Review Board of the University of Southern California (Los Angeles, CA, USA) approved the study protocol.

### Pre-intervention testing

After informed consent is obtained, subjects will receive pre-intervention testing. Pre-intervention testing will include:

#### TMS measurement

A single-pulse magnetic stimulator (Magstim 200^2^) will be used to carry out all TMS studies. A Double Cone 110-millimetre (mm) coil that provides sufficient depth of penetration to reliably stimulate cortical representation of ankle musculature will be used in this study. Subjects from each intervention group will undergo TMS testing both pre- and post-intervention. The primary motor cortex of the hemisphere contralateral to the impaired ankle will be tested. The pre-intervention session will consist of (i) identifying optimal coil position ('hot spot') and motor threshold (MT) for the target leg muscles (tibialis anterior and gastrocnemius), (ii) conducting input-output (IO) curve studies of both muscles, (iii) input-output curve studies will be conducted both with the muscles at rest and with the muscles pre-activated (active contraction condition). The pre-activated condition is required to generate cortical silent period data.

Surface EMG electrodes will be attached over tibialis anterior and gastrocnemius. The EMG signal will be filtered with a bandpass of 1–1000 Hz, amplified, and digitized at 2000 Hz. The data will be graphically displayed and stored for later analysis in 600-millisecond (ms) samples beginning 100 ms before TMS onset (custom written MATLAB module for analog-to-digital sampling (dwaq; dataWizard acquisition, ADW). For the active contraction condition the subject will be asked to perform maximal dorsiflexion and maximal plantarflexion against a load cell, with care taken to minimize contribution from other muscle groups at the thigh or pelvis. The largest of three attempts is taken as the maximum voluntary contraction force (MVC).

To determine optimal TMS coil location and orientation, TMS pulses are delivered while the subject contracts the ankle musculature and at an inter-stimulus interval of 5–10 seconds. The coil is initially placed at the vertex and the optimal scalp position for TMS ('hot spot') is determined by moving the coil a few centimetres (cm) in each direction from this position and observing the site at which the largest motor evoked potential (MEP) is produced at supra-threshold intensities. The initial muscle contraction serves to facilitate induction of MEPs and is used to aid in finding the motor hot spot.

**Figure 2:**
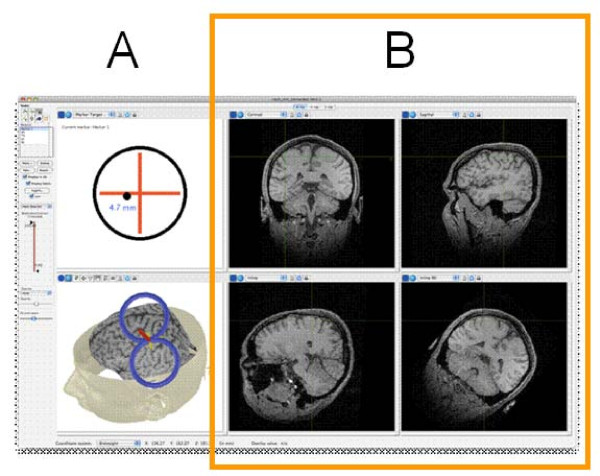
**Screenshot image from Brainsight stereotactic image guidance software used to localize motor hotspot**. A 3-dimensional reconstruction (A, bottom image) of a single T1 magnetic resonance scan of the brain (B) with positioning of the transcranial magnetic stimulation coil. An infrared optical sensor co-registers the position and orientation of the TMS coil relative to small refractory markers on the subject's cranium (A, top image). This provides the investigator with real-time information regarding the brain locus that will receive TMS given a position and orientation of the TMS coil, in order to minimize spatial variability in TMS application. (Image courtesy of Roch M. Comeau, Brainsight, Rogue Industries, Canada.).

Brainsight™ Frameless is a stereotactic image guidance system that will facilitate the positioning of the TMS coil over a subject's brain (Figure 2). With Brainsight, variability in TMS measures taken at different time points will be greatly minimized as the motor 'hot spot' for each muscle is marked on a 3D reconstruction of a standard magnetic resonance image of the brain at the pre-intervention measurement and then the same hot spot location becomes the site of stimulation at the post intervention assessment. A single T1-weighted MRI Scan will be used with the interactive frameless stereotactic system (Brainsight, Rogue Industries, Canada) to guide precise location of the TMS coil and stimulate the appropriate muscle representation in M1. With Brainsight technology a 3D reconstruction of an MRI scan can be made. Subjects will sit in a chair with their head secured. Landmarks on the subject's head will be co-registered with landmarks on the structural MRI to allow tracking of the position of the TMS coil with respect to the underlying cortex. The position of the TMS coil and the subject's head are co-registered from small pieces of refractory material (trackers) placed on them. An infrared optical position sensor monitors the trackers. This information is sent to a computer, which after a calibration procedure displays the position and orientation of the coil relative to the MRI. After the motor 'hot spot' for the tibialis anterior and gastrocnemius muscles are identified, the coil location and orientation is then marked on the anatomic MRI using Brainsight. All subsequent TMS pulses will then be delivered with the coil placed in the same location and orientation as this hot spot.

#### Motor threshold (MT)

MT is defined as the lowest TMS intensity required to elicit a small, clearly discernable MEP at least 50% of the time (50 microvolts [μV] for resting muscles, 100 μV for muscles under active contraction). Within a given individual, MT is stable across TMS sessions which assures that suprathreshold TMS intensities expressed as a percentage of MT can be reliably compared between TMS sessions.[28]

The following procedure for determining active MT will apply for testing the tibialis anterior and gastrocnemius. Active contraction corticomotor excitability data will be collected with the muscles at 10% of the subject's MVC. The coil is oriented and positioned with the handle of the coil pointing backward to induce posterior to anterior current flow across the primary motor cortex. For determination of active MT, the subject makes a steady, minimal background contraction against a load cell (10% of MVC) with the help of audiovisual feedback. TMS pulses are delivered while the subject contracts and at an inter-stimulus interval of 5–10 seconds. The coil is initially placed at the vertex. Optimal coil location for TMS ('hot spot') is determined by moving the coil a few cm in each direction from this position and observing the site at which the largest MEP is produced at supra-threshold intensities. This optimal coil location is then marked on the 3D MRI using Brainsight and the coil is fixed at this location on the scalp using a clamp and external support. TMS intensity is then decreased by 1–2% intervals. TMS MT for the muscle is the TMS intensity where at least 5 of 10 responses generate peak-to-peak MEP amplitudes of at least 50 μV for the resting condition and 100 μV for the active contraction condition. Alterations in corticomotor system excitability will be assessed as changes in MEP and cortical silent period duration (CSP) input-output curve characteristics between pre- and post-intervention time points for the three groups. TMS intensities for this study will be expressed as a percentage of MT.

#### MEP input-output curves

Input-output (IO) curves will be generated by keeping the TMS coil at its optimal hotspot and varying TMS intensity (i.e., input) at 5% intervals from 95% (subthreshold) to 140% (suprathreshold) of MT. At all intensities, 8 single-pulse TMS stimuli will be delivered under each of 2 conditions: (i) at rest and (ii) simultaneous with an active contraction of the muscle (5–10 seconds between stimuli). The order of TMS intensities will be varied randomly.

#### CSP input-output curves

Silent period durations depend largely on the intensity of the TMS stimulus delivered over the 'hot spot' of a pre-activated target muscle. The active muscle contraction condition will be standardized by (i) using audio-visual feedback provided to the subject for consistent levels of muscle contraction (10% MVC) and (ii) triggering TMS discharge when EMG activation is maintained at 10% MVC. As such, CSP durations will be obtained during IO curve studies. At each trial, the duration of the silent period will be calculated as the time between (i) when rectified EMG activity drops below average pre-TMS EMG activity (10% MVC) and (ii) when rectified EMG activity rises above that average pre-TMS EMG activity.

The relationship between the size of the MEP and the duration of the silent period versus stimulus intensity can be illustrated as a sigmoidal shape. As such, measured characteristics of the IO curve will be extracted using the Boltzmann sigmoidal function to fit the data points by the Levenberg-Marquard nonlinear least-mean-squares algorithm.[29] The Boltzmann equation relating the amplitude (MEP) and duration (CSP) of the response with the stimulus intensity has three parameters: the maximum value (i) MEP_max_, the plateau of the relation, (ii) the stimulus intensity (S_50_) required to obtain a response 50% of the maximum, (iii) and the maximum slope parameter.

#### H-reflex

In order to determine whether potential changes in TMS-assessed corticospinal excitability pre- and post-intervention are spinally and/or centrally mediated the Hoffmann (H) reflex will be performed. The H reflex is considered the electrical analogue of the tendon stretch reflex. Since it bypasses the effects of gamma motoneurons and muscle spindle discharge, it can be used as a method for assessing spinal reflex excitability in intact human subjects before and after intervention.[30] The H-reflex will be elicited using a Cadwell Sierra Wave EMG Nerve Conduction Device (Cadwell Laboratories, Kennewick, WA, USA) by selective electrical stimulation of the Ia fibers of the posterior tibial nerve at the popliteal fossa. In response to afferent nerve stimulation, H-waves can then be recorded as MEPs using surface EMG electrodes over the soleus muscle. Such stimulation can be accomplished by using slow (less than 1 pulse/second), long-duration (0.5–1 ms) stimuli with gradually increasing stimulation intensity. As stimulator intensity increases, the peak-to-peak H-wave amplitude gets larger and a direct short-latency motor response (M-wave) also begins to develop. The M-wave indicates that orthodromic motor fibres are now being directly stimulated at higher intensities. Finally, at supramaximal intensity stimulation, the H-wave disappears because antidromic motor stimulation completely blocks descending H-reflex conduction, and only the direct M-wave can be observed.

#### Electrode placement

Subjects are positioned prone with the leg to mid thigh exposed. The active electrode is placed 1/3 of the distance up from the intermalleolar line to the popliteal fossa (or at the base of the bulk of the calf muscle). The reference electrode is located midline of the Achilles tendon. The skin will be marked in order to decrease the variability of pre-post testing electrode placement. The ground is placed between the stimulator and electrodes (i.e., knee cap, femoral condyle) and the amplifier is positioned at the hip away from electrodes.

#### Stimulation procedure

Stimulation will be at the popliteal fossa with the top prong (cathode) placed in the middle of the fossa and superior to the bottom prong. The "Positive" end of the stimulator (anode) is positioned inferiorly toward the ankle. The arrow on the stimulator points in the direction of the current flow. In this case, we want the current flowing proximally toward spine. With the pulse width for stimulation set at 1 ms, stimulation is started at a low intensity. Intensity is increased in 1 milliampere (mA) increments. The stimulator delivers pulses at regular intervals (pulse width = 1000 ms) to the Ia afferents. Intensity is gradually increased until an H wave appears. At this point, intensity is increased at 1–2 mA per discharge until the H wave reaches maximum, starts to decrease and the M-wave appears. Intensity is now increased in larger increments (5–10 mA) until the H wave completely disappears and the M-wave reaches a maximum.

#### Ankle range of motion (ROM) measurement

Following the second TMS MT measurement, all subjects will receive 2 different ankle ROM measurements. In the first measurement, subjects will lay prone on a padded table. A single blinded and standardized examiner will measure ankle dorsiflexion ROM with the knee fully extended and then flexed to 90 degrees using a 15.24 cm goniometer in a standard manner.[19] This measurement of ankle ROM demonstrates strong test-retest reliability with knee both flexed (ICC = .97) and extended (ICC = .98).[19] A knee to wall measurement also will be taken.[31] Standing with hand support on the wall, with affected lower extremity anterior and the unaffected lower extremity posterior. Subjects were required to move the knee directly forward to a vertical line on the wall. No attempt will be made to control the height of the medial longitudinal arch during this measurement. For subjects who are unable to touch their knee to the wall (1), the distance from the wall to the anterior aspect of the knee will be measured and recorded in centimeters as a negative value. Subjects who can touch the knee to the wall will move the foot back until the knee could just touch the wall (2). The distance from the wall to the great toe will be recorded in centimeters as a positive value. The knee to wall measurement of ankle ROM also demonstrates strong test-retest reliability (ICC = .97).[31]

#### Lower extremity functional behavior measurement

Following the ankle ROM tests, all subjects will receive a measurement of lower extremity functional behavior involving a star balance excursion test (SBET) conducted on a single limb. The SBET is a clinical test of dynamic balance.[32] Subjects will assume unilateral stance in the center of a grid marked circumferentially in 45-degree increments. After a learning trial consisting of 6 repetitions in each of the 8 test directions[33], subjects will complete 3 repetitions of single limb squat reach. Two trials will be completed: 1 trial each with the subject standing on the affected and unaffected limbs. Test directions include anterior, lateral, anterolateral, posterolateral, posterior, medial, anteromedial, and posteromedial. The evaluating therapist will record the distance achieved between the stance toe and the contacting portion of the reaching extremity for 3 repetitions in each direction. Fifteen seconds of rest will be provided between trials. Repetitions will be excluded if the subject (1) is unable to maintain weightbearing during the trial; (2) lifts the stance foot; (3) loses balance; or (4) does not maintain the hold or start positions for 1 second. This test demonstrates good reliability (ICC = .67–.97), and demonstrates discriminative validity between non-disabled individuals and patients with chronic ankle instability.[32,34]

### Intervention

**Figure 3:**
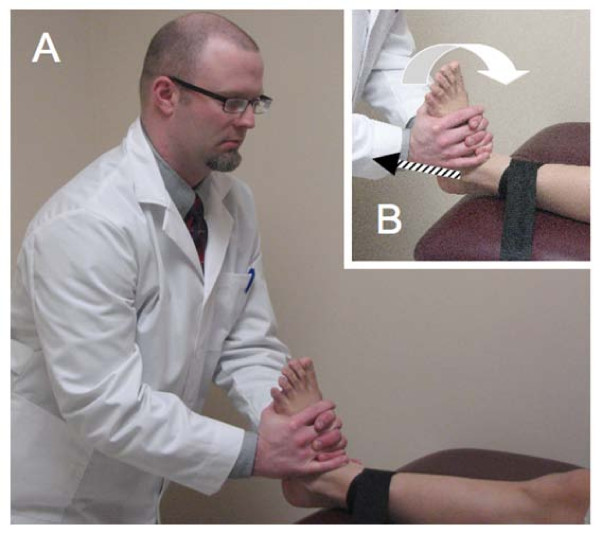
**Ankle high-velocity low-amplitude, slow velocity, and control interventions under study**. With the subject in a supine position on a treatment table and the lower extremity of interest stabilized to the table with a belt (A), the treating investigator will grasp the foot of interested with the thenar eminences on the foot's plantar surface (B) and induce passive dorsiflexion to end range (B; open arrow). Iatrogenic force will be provided along the long axis of the tibia in the intervention groups. (B; hatched line) In the control group, the treating investigator will maintain passive dorsiflexion (B; open arrow) for the duration of 1 deep inhalation and exhalation by the subject rather than induce an iatrogenic force.

Subjects will be randomized into 3 groups after pre-participation screening is completed, informed consent is obtained, and baseline measures are recorded. The first group (n = 9) will receive an HVLA procedure directed to the talocrural joint (Figure 3). With the subject in a seated position on a treatment table and the lower extremity of interest stabilized to the table with a belt, a standardized licensed physical therapy (treating therapist) will grasp the foot of interested with the thenar eminences on the foot's plantar surface. A thrust will be delivered parallel to the long axis of the subject's lower leg after the treating therapist induces passive ankle dorsiflexion to end range. The second group (n = 9) will receive a slow procedure directed to the talocrural joint. Traction will be delivered to the talocrural joint at the treating therapist's second perception of tissue resistance in 3 bouts of 30-second holds, separated by 10 seconds of rest. The third group (n = 9) will receive the manual therapy control intervention. This will consist of the same patient and clinician preparation for the mobilization/manipulation techniques. However, the treating therapist will simply maintain passive DF ROM for the duration of 1 deep inhalation and exhalation by the subject rather than induce an iatrogenic force.

### Post-intervention testing

Immediately following intervention, subjects will receive a final administration of ankle ROM tests, lower extremity functional behavior test, TMS, and H-reflex measurements. After post-intervention testing, subjects will be discharged from the study.

### Data Analysis

#### TMS measurements

All TMS/EMG data will be analyzed off-line with a customized MATLAB (Mathworks, MA, USA) software tool for analysis of time-series data (dataWizard[35]). Each TMS trial will be analyzed for both MEP and cortical silent period (CSP) duration. At low intensities that did not create a MEP, the maximal peak-to-peak envelope within the time window for typical MEPs will be recorded as the MEP value for that trial. The 8 MEPs occurring at each level of intensity will be averaged. The CSP duration will be defined as the time between TMS and the first return of rectified EMG activity of at least 50% of pre-TMS background activity following a period of sustained silence. The CSP duration will be calculated relative to TMS onset because at lower intensities MEPs often will be absent while CSPs can be detected. When no CSP can be discerned, CSP duration will be marked as 0 msec. Eight CSP duration values will be averaged for each subject. The relationship between average MEP amplitude and CSP data (output) and TMS intensity (input) will be fitted to a sigmoid curve.[29,36] These sigmoid functions will then be characterized by 3 parameters: maximal amplitude, maximal slope, and the midpoint intensity where amplitude is half maximum. The ratio of the largest H and M wave amplitudes also will be calculated (H/M ratio). If the ratio remains the same pre and post ankle thrust then any change in corticomotor excitability assessed through TMS would be considered centrally mediated.

#### Functional behavior and ROM measurements

SBET measurements will be indexed to lower limb length as measured from anterior superior iliac spine to medial malleolus prior to data analysis. Two-sample t-tests with appropriate correction will be used to determine the statistical significance of differences in pre- and post-intervention ankle DF ROM, SEBT performance, and MEP/CSP data. Test-retest reliability and minimum detectable change will be determined for the 2 baseline measurements of MEP/CSP data.[37,38] Pearson correlations with appropriate statistical correction will be used to test the significance of associations among ankle DF ROM, SBET measurement, and TMS/EMG variables within subjects.

For all TMS, ROM, and lower extremity functional behaviour variables, two-factor multivariate analysis of variance with repeated measures for time will be used to determine the effects of repeated baseline testing and the intervention under study on SBET performance and ankle ROM measurements. Significant main effects will be reported if there are no significant interactions. If significant interactions are found, then one-way analysis of variance will be completed for each variable. Post-hoc testing will be performed as necessary with appropriate correction to determine the significance of pairwise comparisons. Pearson correlations with appropriate statistical correction will be used to test the significance of associations among ankle DF ROM, SBET measurement, and TMS/EMG variables within subjects.

## Discussion

This study protocol describes one of the first reported feasible study methodologies to date that is designed to identify potential neuromotor changes associated with manual therapy procedures directed to the appendicular skeleton. We believe the large muscles associated with the talocrural joint and ability to localize the procedure to the talocrural joint will better elucidate potential neuromotor effects of manual therapy procedures than the studies to date that have focused on spinal procedures. Further, we will be able to compare the relative effect of treatment speed on potential neuromotor effects of manual therapy procedures in the context of a controlled study. Also, this study will provide the ability to make inferences regarding the functional relevance of changes in corticospinal excitability through the use of measurements of lower extremity range of motion and functional behavior. These characteristics will allow this study's data to further our understanding of appropriate clinical management of individuals with many forms of musculoskeletal dysfunction.

## Competing interests

The authors declare that they have no competing interests.

## Authors' contributions

BEF, TED, KK, and AW contributed to the design of this study protocol. TE Davenport provided research funding.

## Pre-publication history

The pre-publication history for this paper can be accessed here:


